# Understanding Microglia in Mesocorticolimbic Circuits: Implications for the Study of Chronic Stress and Substance Use Disorders

**DOI:** 10.3390/cells14131014

**Published:** 2025-07-02

**Authors:** David B. Nowak, Juan Pablo Taborda-Bejarano, Fernando J. Chaure, John R. Mantsch, Constanza Garcia-Keller

**Affiliations:** 1 Department of Pharmacology and Toxicology, Medical College of Wisconsin, Milwaukee, WI 53226, USA; dnowak@mcw.edu (D.B.N.); jtaborda@mcw.edu (J.P.T.-B.); fchaure@mcw.edu (F.J.C.); 2 Medical Scientist Training Program, Medical College of Wisconsin, Milwaukee, WI 53226, USA

**Keywords:** drug misuse, addiction, chronic stress, psychostimulants, microglia, 3DMorph, IMARIS, neuronal plasticity, cocaine

## Abstract

Exposure to chronic stress creates vulnerability to drug misuse and presents a barrier to sustained recovery for many individuals experiencing substance use disorders (SUDs). Preclinical literature demonstrates that stress modulates psychostimulant intake and seeking, yet there are wide gaps in our understanding of the specific mechanisms by which stress promotes brain changes that may govern addiction-related behaviors. Recent data suggest that microglia, innate immune cells in the central nervous system, are highly responsive to chronic stressors, and several mechanistic links have been explored highlighting the critical role microglia play in stress-related brain adaptation. Importantly, psychostimulants may engage similar microglial machinery, which opens the door for investigation into how microglia may be involved in shaping motivation for psychostimulants, especially in the context of stress exposure. The aims of this review are threefold: 1. Offer a brief overview of microglial biology in the adult brain. 2. Review current methods of interrogating microglial function with a focus on morphometric analyses. 3. Highlight preclinical research describing how microglia contribute to brain changes following chronic stress and/or psychostimulant exposure. Ultimately, this review serves to prime investigators studying the intersection of stress and SUDs to consider the relevant impacts of microglial actions.

## 1. Introduction

Chronic exposure to uncontrollable stressors is associated with an increased risk for the development of mood, trauma, psychotic, and addiction-related disorders [1]. The neurobiological mechanisms that drive each of these conditions are likely heterogeneous; however, life stress exposure serves as a common thread for many individuals facing neuropsychiatric disease. Substance use disorders (SUDs) are no exception. Individuals who engage in drug use patterns that conflict with their wellness goals reference both acute and chronic stress exposure as a contributing factor for persistent misuse or relapse after a period of abstinence [2,3]. Therapies intending to mitigate stress-related processes contributing to drug misuse have not achieved wide clinical success [4,5,6]. Therefore, continued study is needed to parse out the relevant neurobiological underpinnings of comorbid stress-related disorders and substance misuse to identify effective therapeutic interventions. In this review, we focus on the neuroimmune consequences of stress and psychostimulant exposure, highlighting the role of microglia in the emergence of behaviorally relevant neuronal plasticity in limbic nodes. The scope of this review is generally limited to the nucleus accumbens (NAc), ventral tegmental area (VTA), and prefrontal cortex (PFC), but attention is paid to other limbic structures, including the amygdala and hippocampus, when appropriate.

Microglia, first identified and described a century ago, are a population of tissue-resident macrophages found in the brain and spinal cord. They constitute approximately 10-15% of the total cells in the central nervous system (CNS) and represent the most prevalent mononuclear phagocytes in this compartment. Lineage tracing studies demonstrate that microglia migrate to the brain from the extra-embryonic yolk sac during early development and engage in carefully balanced self-proliferation and apoptosis, as needed, in adulthood [7,8]. Closely related cells, including monocytes and border associated macrophages (BAMs, also referred to as brain or CNS-associated macrophages) can be found among microglia in the CNS and share key molecular markers including ionized calcium-binding adaptor molecule 1 (IBA1), CD11b/c, and Cx3cr1 (fractalkine receptor). To distinguish microglia from related macrophage populations, TMEM119+ (transmembrane protein 119) and/or P2Y12R+ (purinergic P2Y receptor) have been used as markers when technically feasible. Approaches that afford specific labeling of microglia may offer greater insight into the functions of these cells in different health and disease contexts, as they may serve unique functions compared to neighboring immune cells [9,10]. For a comprehensive review of microglial nomenclature, see [11].

Throughout the past decade, remarkable advancements in our understanding of microglia functions unfolded. Of note, neurodevelopmental studies elucidated a role for microglia in activity-dependent remodeling of the visual system [12,13,14,15]. The idea that microglia can directly influence neuronal structure and function in the developing nervous system sparked significant interest in understanding how microglia in the adult brain affect neuronal activity and behavior. Experiments investigating microglial function in the context of immune defense, aging/neurodegeneration, neurovascular function, neuromodulation, and the response to ischemic or traumatic brain injury significantly broadened our understanding of microglial influence in the brain. Although microglia function as bona fide immune cells, possessing receptors intended to identify “non-self” threats to homeostasis, they also utilize a diverse array of receptors intended for endogenous signals, allowing them to integrate molecular cues from neighboring neurons and glial cells. Through a dynamic interplay with neighboring CNS cells, microglia can exert a functional influence on brain networks, importantly, in the absence of a canonical inflammatory insult [16,17].

Recent studies highlighted microglia as a crucial bridge between stress signaling and alterations in neuronal structure and function that are correlated with depression- and anxiety-like behaviors. A goal of this review is to explore the possibility that microglia may be important to the emergence and/or maintenance of stress-associated behaviors, including psychostimulant misuse.

## 2. What Is the Role of Microglia in the Adult Brain?

***Innate immune function:*** Microglia, similar to other tissue-resident macrophages, express a battery of receptors, which allows them to identify and engage “non-self” materials. Innate immune function is punctuated by a broad defense strategy, whereby immune cells recognize pathogens or damage-associated molecular patterns using pattern recognition receptors (PRRs). Once pathogens are detected, microglia can release pro-inflammatory cytokines, such as TNF-α, IL-1β, and IL-6, with the intention of recruiting additional immune cells (T cells, neutrophils, etc.) to a region of concern. Additionally, microglia are well-suited to clear entire cells or debris via phagocytosis. Phagocytosis may be initiated via the complement system, where complement component 1 (C1q) is deposited or uncovered on material that is destined for elimination. Microglia express complement receptor 3 (C3R), which detects C1q and facilitates phagocytic elimination. Once ingested, microglia employ lysosomal degradation to break down engulfed products. A lysosomal protein, CD68, has been widely used as an indicator of microglial phagocytic activity in histological analyses [18]. 

***Microglia development and survival:*** Colony-stimulating factor 1 receptor (CSF1R) belongs to the class III transmembrane receptor with tyrosine kinase activity expressed in macrophages and microglia in the brain [19]. CSF1R is posited to be the major regulator of microglial development, proliferation, differentiation, and survival, but also regulates homeostatic microglial function [20,21]. There are two natural CSF1R ligands, colony-stimulating factor 1 (CSF1) and interleukin 34 (IL34), both of which are differentially expressed in the developmental and adult stages in the brain. Ligands induce the homodimerization of CSF1R and activation of the activation downstream signaling pathway. CSFR1 −/− null mice do not survive into adulthood and show almost complete microglia depletion [22]. Mice with CSF1R haploinsufficiency exhibit loss of microglial homeostasis that has been associated with loss of presynaptic surrogates, extracellular matrix structure, and postsynaptic markers [23]. 

***Diverse array of immune receptors:*** Microglia express a variety of pattern recognition receptors (PRRs) that detect pathogen-associated molecular patterns (PAMPs) and damage-associated molecular patterns (DAMPs). These PRRs include Toll-like receptors (TLRs); NOD-like receptors (NLRs); receptors for nucleic acids, and C-type lectin receptors (CLRs) [24]. Additionally, microglia express several receptor families involved in the phagocytosis or endocytosis of apoptotic cells, protein aggregates, and lipoprotein particles. These include scavenger receptors, LDL receptors, and receptor tyrosine kinases. Microglia also express chemokine receptors including CX3CR1 and CXCR4, as well as integrins such as CD11b and CD11c. These receptors regulate the migration and positioning of microglia within the CNS and enhance their ability to engage target cells for phagocytosis and elimination [25].

***Soluble factor secretion and neuronal remodeling:*** Although responding to inflammatory stimuli (i.e., canonical immune threats) is intrinsic to microglial identity, the role of microglia in maintaining homeostasis in the adult brain is equally significant. Moreover, cytokine secretion by microglia is not necessarily indicative of a binary transition from a homeostatic to inflammatory phenotype (e.g., M1 vs. M2 states). Instead, soluble factor secretion contributes to a finely tuned neuroimmune communication system, including the regulation of synaptic function. Importantly, the release of cytokines, such as Tnf-α, has been implicated in mediating cellular and behavioral effects of acute stress exposure through modulation of synaptic strength [26]. Microglia-derived IL-1β is another inflammatory cytokine that exerts local influence on neuronal function, especially in the context of pathology and aging [27]. 

Alternatively, neurons may secrete cytokines, including IL-33, that signal via the IL-33 receptor found on microglia and have been shown to be crucial for plasticity related to the expression of fear memories [28]. Growth factors such as BDNF can also be secreted by microglia to facilitate learning or experience-dependent synaptic remodeling [29]. A recent study by Woodburn et al. adds that microglia-derived BDNF may be important for maintaining cortical synapse density despite exposure to chronic mild stress [30]. Prior to this study, it was unclear if BDNF from microglia played a meaningful role in preserving dendritic complexity in the face of stress. This example further illustrates the nuanced role of microglia in promoting both susceptibility and resilience to neurobiological effects of stress. 

***Neurochemical sensation:*** In addition to immune-related receptors, microglia express receptors for endogenous signals such as neurotransmitters and bioenergetic substrates. One mechanism by which microglia can sense ongoing neuronal activity is through monitoring of purinergic signals. These molecules include ATP, ADP, and adenosine (ADO), which are sensed by P2Y and P2X receptors. P2Y12R is a Gi-coupled G-protein-coupled receptor (GPCR) that is involved in microglial chemotaxis and inflammatory signaling while serving as a putative microglia-specific (tissue resident macrophage) marker in the brain [31]. Recently, the P2Y12 receptor has been shown to modulate levels of intracellular Ca^2+^ in microglia, which is associated with modulation of cortical norepinephrine (NE) signaling [32]. The P2X4 and P2X7 receptors are ionotropic receptors that lead to calcium influx upon activation [33]. Several studies suggest that ATP is co-released with excitatory neurotransmitters such as glutamate [34] or dopamine [35]. Periods of sustained excitatory neurotransmission, such as during stress exposure or psychostimulant use, may recruit microglia via purinergic signaling. Interestingly, stimulation of the P2X7 receptor in microglia also leads to increased signaling by the endocannabinoid, 2-arachidonoylglycerol (2-AG) [36]. Microglia express receptors for glucocorticoids, stress-related peptides (corticotropin releasing factor; CRF), glutamate, γ-aminobutyric acid, NE, acetylcholine, and endocannabinoids (CB1 and CB2), enabling them to not only participate in the stress, but receive signals from paracrine or endocrine signals as well [37].

***Baseline sex differences:*** As mentioned above, microglia express a wide variety of receptors, enabling sensitivity to a dynamic biochemical environment. Receptors that bind hormones and neuropeptides that exhibit sexually dimorphic regulation (e.g., estrogens, androgens, and progesterone) are important for informing sex-specific microglial function. The extent to which specific hormone receptors drive sex differences in microglial function is a topic of ongoing research. Primary microglia cultures served as invaluable resources for studying how microglia respond to environmental changes. However, culture-derived insights regarding microglial functions often failed to align with in vivo observations. Expression of estrogen receptors (ERs) differs between these cell lines and among primary cultures. For example, BV-2 cells express ERβ, but not ERα, while N9 cells express both classical estrogen receptors [38]. Histological and transcriptomic studies in rodents determined that microglia exhibit baseline differences in male and female animals [39]. Guneykaya and colleagues observed a significant elevation in microglial cell density (Iba1+ cells) in male vs. female adult mice [40]. The authors observed the referenced increase in cell density in the cortex, hippocampus, and amygdala, while reporting no difference in cell density in the striatum or cerebellum. Additionally, they reported that male microglia displayed an increased soma size in the cortex, hippocampus, and amygdala. Although the functional implications of these morphological differences remain undetermined, acknowledgement of baseline differences in microglial properties should inform later analysis when observing the microglial response to environmental or pharmacological manipulations.

## 3. Comparing Histological Methods for Interrogating Microglia Function After Pharmacological and Environmental Exposure

***Comparison of transcriptomic, proteomic, and morphometric methods for microglial analysis:*** Recent literature emphasized the importance of appropriately categorizing microglial states with data collected in different assays [11]. We will mention three widely used techniques that provide functional information about the microglial state: transcriptional analysis, protein analysis (proteomics), and morphological analysis. Some transcriptional assays that have been used to study microglia in the previous literature [26,41,42,43,44] are whole- and single-cell RNA sequencing, quantitative PCR, and RNA in situ hybridization (RNAscope). RNA-based techniques are useful for understanding the functional state of microglia at the time of tissue collection. However, uncertainty that mRNA has been successfully translated into functional proteins limits the ability to draw an accurate conclusion about microglial state. Proteomic analysis, on the other hand, may provide more accurate information about the functional state of microglia at the time of tissue harvest. Techniques used to quantify protein levels in microglia include flow cytometry- and Western blot analysis [45,46,47,48,49]. The advantages of protein-based analyses are offset by limitations related to the availability and affinity of antibodies needed to tag proteins of interest, often making it challenging for experimenters to rely on these techniques.

Morphological analyses long served as a proxy for assessing microglial function, offering visual insights into cellular state and activity. Traditionally, ramified microglia—with highly branched processes—have been associated with a homeostatic or surveillant phenotype, whereas amoeboid microglia—characterized by a more rounded, process-devoid morphology—are interpreted as pro-inflammatory. However, emerging evidence suggests that microglial morphology exists along a spectrum that extends beyond this binary classification. Microglia can assume a variety of intermediate forms that may not align neatly with their functional state, and some functions may occur independently of overt morphological changes [11]. Thus, while morphology alone cannot definitively indicate microglial activity or phenotype, it remains a valuable tool for assessing spatial and temporal changes in response to pharmacological and environmental stimuli. Region-specific shifts in microglial morphology can yield important clues about underlying neural processes, particularly in contexts such as stress exposure or drug administration.

To conduct morphological studies, researchers commonly rely on immunohistochemical labeling of microglia using markers such as IBA-1, a calcium-binding protein expressed in both microglia and macrophages. IBA-1 is widely used because it is distributed throughout the microglial cytoplasm, allowing for detailed visualization of cell shape and process complexity [50]. Other markers—including CD11b, CX3CR1, and CD68—are also employed, though they lack microglial specificity. More selective markers, such as TMEM119 and P2Y12, offer greater precision in distinguishing microglia from other brain-resident macrophage populations. Additionally, transgenic reporter lines (e.g., *CX3CR1*-GFP, *TMEM119*-GFP, and *Sall1*-GFP) have been developed to facilitate in vivo or ex vivo imaging, though most of these tools are limited to mouse models [51,52,53]. In rats, the only available option is a cre-dependent *CX3CR1*-ERT2 line, limiting genetic imaging strategies in this species. Despite these constraints, IBA-1 continues to be a robust and widely accepted marker for microglial morphological analysis across a range of mammalian species [54].

Recent advancements in morphological analysis platforms, such as IMARIS and CellSelect-3DMorph [55], significantly improved the ability to quantify microglial structure in three dimensions with greater precision and throughput. While these tools vary in terms of processing speed, user accessibility, and analytical capabilities, both are effective in detecting alterations in microglial complexity and spatial territory following experimental interventions. Despite its limitations, morphological profiling remains a vital and widely accessible component of this multimodal approach. To achieve a comprehensive understanding of microglial state, it is essential to integrate morphometric data with transcriptomic and proteomic analyses. As research progresses, the development of more refined, cell-specific, and regionally targeted techniques will be key to elucidating the intricate neuroimmune processes that influence behavior.

## 4. Microglia Mediate the Effects of Chronic Stress on the Brain

**Figure 1:**
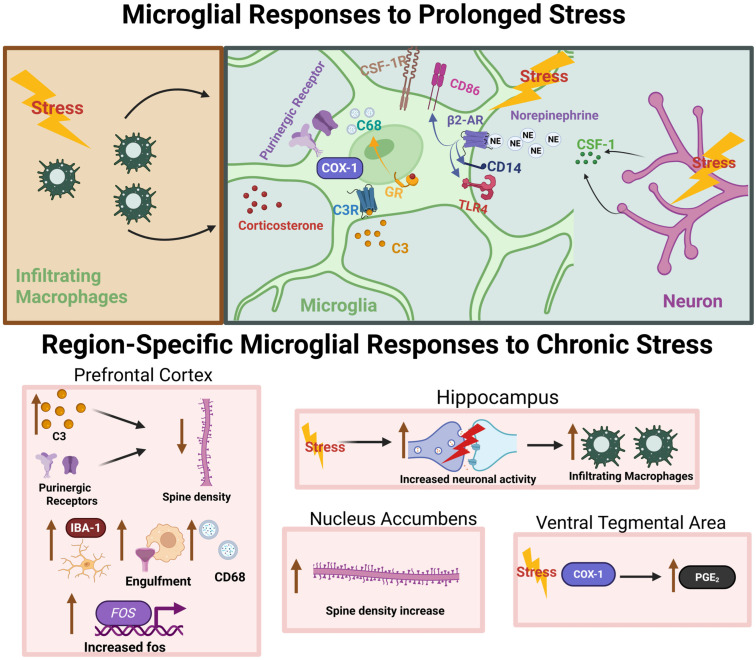
**Overview of chronic stress effects on microglia across brain regions.** The upper panel of the figure illustrates the primary effects of chronic stress on microglia, highlighting three distinct microglial cells exhibiting stress-induced alterations. In contrast, the lower panel expands the view to show the broader impact of chronic stress on an entire brain region, contextualizing microglial changes within the larger neuroanatomical environment. On the left side of the upper panel, chronic stress is shown to induce infiltration of peripheral macrophages across the blood–brain barrier. These cells demonstrate increased chemotaxis toward hyperactive neurons [58,59]. On the right, stress-induced neuronal release of colony-stimulating factor 1 (CSF1) is depicted, a mechanism known to contribute to synaptic plasticity changes in the prefrontal cortex (PFC) [60]. The central part of the upper panel outlines key intracellular and receptor-mediated processes in microglia following chronic stress. Two primary stress-related mechanisms are emphasized: (1) corticosterone diffusion into microglia, where it binds nuclear glucocorticoid receptors (GR), triggering engulfment of synaptic components within CD68 lysosomes (yellow arrow) [61], and (2) norepinephrine activation of β2-adrenergic receptors, leading to upregulation of pro-inflammatory surface markers, including CD14, TLR4, and CD86 (purple arrows) [62]. Additional chronic stress effects are mediated through other pathways, including CSF1R (the receptor for neuronal CSF1), purinergic receptors, COX-1 signaling, and complement component 3 (C3) signaling [49,63,64]. The lower panels focus on the region-specific microglial effects of chronic stress. In the PFC, stress-induced alterations include purinergic and complement C3 signaling-dependent dendritic atrophy [49,63], increased IBA1+ cell counts, elevated CD68 expression, enhanced neuronal engulfment, and increased *fos* expression [63]. In the nucleus accumbens (NAc), chronic stress promotes increased dendritic spine density. In contrast, the ventral tegmental area (VTA) shows COX-1-dependent elevations in prostaglandin E2 (PGE2), linked to social deficits under stress [64]. Finally, stress-induced recruitment of monocytes and non-microglial macrophages is primarily observed in the hippocampus. Collectively, this figure summarizes the key mechanisms and regional microglial responses described in the current literature regarding the effects of chronic stress on the brain. Figure made using BioRender.com.

The stress response is initially adaptive; however, mobilization of stress-related machinery comes at a high bioenergetic cost, especially when stressor resolution is protracted or unpredictable [56]. Preclinical models of chronic stress exposure in rodents demonstrate stress-induced alterations in dendritic complexity in mesocorticolimbic nodes that are accompanied by deficits in circuit function [57]. Glucocorticoids and stress-related peptides, such as corticotropin-releasing factor (CRF; also known as corticotropin-releasing hormone, CRH), contribute to stress-related changes through direct action on neurons. However, recent studies suggest that microglia are also receptive to stress signaling and may serve as an effector of neuronal adaptations at the outset and throughout a bout of chronic stress (See Figure 1 for overview of chronic stress effects on microglia).

The relationship between chronic stress exposure and immune system action has been characterized in preclinical literature [65,66,67]. Engagement of the peripheral immune system can promote the generation of an inflammatory milieu in the body as well as the brain since many inflammatory cytokines can cross the blood–brain barrier. Microglia can both respond to and generate an inflammatory reaction to prolonged stress exposure [68].

The underlying mechanisms of stress-induced neuronal plasticity are incompletely understood, and, crucially, may involve a careful interplay among neuronal and glial cells [69]. Recent investigations of microglial contributions to stress-induced neuronal remodeling demonstrated that microglial action is intimately related to the structural and functional sequalae of chronic stress [68,70,71,72,73]. Importantly, targeting microglia-specific receptors or processes can rescue or prevent the negative consequences of stress exposure [30,49,63,74,75,76,77,78,79,80,81,82,83]. This section will highlight key studies demonstrating the role of microglia in chronic stress-induced mesocorticolimbic remodeling. 

***Stress and microglia in the medial prefrontal cortex:*** Multiple laboratories demonstrated that chronic stress exposure precipitates marked changes in dendritic architecture in several mesocorticolimbic nodes, including the PFC [84]. Increasing evidence indicates that microglia in the PFC are necessary for changes in neuronal structure and anxiety or depression-like behaviors that occur after chronic stress [73,76]. Work by Wohleb et al. demonstrates that both patients diagnosed with MDD examined post-mortem and rodents exposed to a chronic unpredictable stress paradigm exhibit increased expression of neuronal colony-stimulating factor 1 (CSF1) in PFC [60]. CSF1 is a signaling factor necessary for the survival of microglia, as inhibition of the CSF1 receptor leads to microglial clearance [85].

Increased CSF1 ligand signaling appears to be an integral link between chronic stress exposure and the emergence of dendritic atrophy and functional deficits in the PFC. Ultimately, knockdown of CSF1 expression in PFC neurons prevents the structural and functional consequences of chronic stress exposure in rodents [60]. Notably, the CSF1R has two endogenous ligands, CSF1 and IL-34. Little is known about the effects of chronic stress on IL-34 signaling, although it has recently been implicated in directing microglial function in the context of neuroinflammation and aging [86].

CSF1 signaling may be involved in microglial migration, an essential step in immune cell recruitment. However, microglia also possess other receptors that respond to neuronal cues in the PFC. Purines, namely adenosine diphosphate (ADP) and ATP, function as chemotactic signals in the brain. Microglial P2X and P2Y receptors, ionotropic and metabotropic purinergic receptors, respectively, bind endogenous purines and facilitate microglial chemotaxis. Recently, Bollinger et al. demonstrated that microglial P2Y12R is necessary for prefrontal cortical changes associated with chronic stress exposure in mice, including an increase in microglial inclusion of neuronal materials [49]. Although direct phagocytosis, or engulfment, of synapses by microglia presents an exciting explanation for the observed decrease in cortical spine density and increased engulfment of neuronal material, direct evidence of this phenomenon is lacking [87].

The complement system, a cornerstone of immune-mediated degradation, plays a role in cortical remodeling following chronic stress exposure. Wang et al. show that complement C3 is upregulated following chronic social defeat stress (CSDS) in concert with a reduction in both pre- and post-synaptic components [63]. This stress paradigm resulted in an increased number of Iba1+ cells, CD68 volume, and synaptic component engulfment by microglia. Moreover, CSDS resulted in C3-dependent attenuation of medial PFC connectivity measured via resting-state fMRI. Genetic C3 knockout prevented the effects of CSDS on synaptic architecture and function as well as microglial engulfment activity. These data collectively argue for the role of complement C3 in facilitating the microglia-dependent consequences of CSDS exposure in PFC.

Furthermore, a recent investigation by Tillmon and colleagues described increased complement C3 deposition in the medial PFC of mice after chronic corticosterone treatment, chronic unpredictable stress, and chronic restraint stress [61]. The increased complement deposition was localized to layers II/III and no differences were observed in layer V. Chronic corticosterone treatment promoted a reduction in synapse density measured via colocalization of VGlut2/PSD-95 markers, but not VGlut1/PSD-95 or VGAT/Gephyrin, suggesting a decrease in cortico-thalamic excitatory synapse density. Critically, complement signaling was determined to be necessary for this reduction in synapse density as C3 knockout mice were unaffected by chronic corticosterone exposure. VGlut2 engulfment by microglia (determined by volume of VGlut2 within CD68^+^ lysosome divided by volume of Iba1^+^ cell) was found to be increased after chronic corticosterone. C3 knockout also protected mice exposed to chronic corticosterone from deficits in the PFC-dependent temporal object recognition task. Single-cell RNAseq analysis revealed a unique transcriptional signature for cortical microglia following chronic corticosterone administration highlighted by the emergence of an *Apoe*^high^ state. This study demonstrates the relevance of a pro-engulfment microglial state due to elevated glucocorticoid signaling resulting in behaviorally relevant PFC synapse remodeling.

***Stress and microglia in the nucleus accumbens (NAc) and ventral tegmental area (VTA):*** Investigations of motivated behaviors often center on the NAc due to its linkage with effector nodes such as the ventral pallidum. Upstream influences by PFC, the hippocampus, and basolateral amygdala play an important role in NAc function through excitatory glutamatergic signaling. Moreover, the NAc is a main dopaminoceptive hub that not only integrates signaling from VTA dopamine neurons, but also sends efferent projections to multiple VTA neuronal subtypes [88]. The contributions of microglia to the effects of chronic stress in the nucleus accumbens and VTA received less attention relative to the cortical and other limbic regions mentioned above. Nevertheless, data suggest that microglia in these regions react to chronic stress exposure and may be consequential for the development of behavioral deficits in stress-related conditions.

Chronic stress exposure leads to increased dendritic spine density in the NAc and is associated with the emergence of depression- and anxiety-like symptoms [89,90]. Bessa et al. demonstrated that rats exposed to a chronic mild stress protocol develop anhedonia-like qualities measured using a sucrose preference task that are accompanied by hypertrophy of medium spiny neurons in the NAc [89]. The authors showed that changes in neuronal morphology could be reversed by treatment with traditional antidepressants (SSRIs). Work by Gaspar and colleagues demonstrated that short- and long-term exposure to unpredictable chronic mild stress leads to altered microglial and neuronal morphologies [90]. These studies also elucidated a sex-dependent effect of stress on changes in neuronal morphology following stress exposure. Male rats showed hypertrophy of medium spiny neurons in the NAc following stress as well as depressive- and anxiety-like symptoms. However, female rats failed to show changes in medium spiny neuron morphology but exhibited anxiety-like behavior after either short- or long-term unpredictable chronic stress. Chronic stress failed to elicit a significant difference in behavioral screens for depressive behaviors including sucrose preference and forced swim tests. These findings highlight the divergent effects of stress relative to the brain region examined and between sexes. Future studies are needed to understand how biochemical context promotes either hypertrophy or hypotrophy of neurons in a region-specific and sexually dimorphic manner. 

The mesolimbic circuit includes robust dopaminergic projections from the VTA to the striatal and cortical areas. Acute stress may transiently increase VTA dopamine neuron excitability, while chronic stress leads to a reduction in dopaminergic tone [91]. A study by Tanaka and colleagues found that microglia contribute to altered VTA dopamine signaling through the release of prostaglandin-E2 in mice following CSDS [64]. In this study, the authors demonstrated that CSDS induced social avoidance behavior following exposure to a social defeat paradigm in male mice. In the mice that underwent defeat, there was a significant increase in brain PGE_2_ levels. The investigators then showed that deletion of cyclooxygenase-1 (COX-1), an enzyme responsible for generating prostaglandins, was necessary to produce social deficits following stress exposure. Furthermore, COX-1 was shown to be localized to Iba1+ cells in the VTA. EP1, a PGE_2_ receptor, was found to be necessary for the development of social avoidance behaviors following stress. The authors also reported that EP1-dependent reductions in VTA-medial PFC dopamine signaling may mediate the neuroendocrine and behavioral hallmarks of chronic stress exposure in this model. These studies demonstrate that microglia, through the production of PGE_2_ via COX-1, regulate VTA dopamine dynamics by signaling via EP1.

***Stress and microglia in the amygdala and hippocampus:*** Chronic stress has widespread effects on neural circuitry which are not limited to prefrontal regions. Limbic structures, including the hippocampus and amygdala, are remodeled by stress, correlating with relevant behavioral alterations. Key discoveries of microglial involvement in stress-related remodeling have been observed in these regions.

The hippocampus is a topographically diverse structure with a high degree of functional segregation. The dentate gyrus is particularly important because it has been implicated as a main site of adult hippocampal neurogenesis (AHN). Many antidepressant pharmacotherapies promote increased AHN, and preclinical studies demonstrated that forcible induction of AHN can attenuate the effects of chronic corticosterone exposure on depression-like behaviors [92]. In mice, chronic stress exposure leads to altered microglial function and is associated with reduced hippocampal neurogenesis [93]. For a review of microglial involvement in AHN, see [94].

Repeated social defeat stress leads to altered neuronal activity and microglial morphology in several limbic nodes, including the hippocampus and amygdala [62]. A study by Wohleb and colleagues shed light on the interplay among beta-adrenergic signaling and CD11b+ cells after social defeat stress [62]. Histological staining for *fos*, an immediate early gene used as a marker of neuronal activity, showed marked increases in neuronal activity in the PFC, lateral septum, bed nucleus of the stria terminalis (BNST), and paraventricular nucleus of the hypothalamus (PVN) after stress exposure. Important for this study, the authors showed that beta-adrenergic receptor antagonism using propranolol was sufficient to prevent the increase in the number of *fos*-positive cells as well as increases in anxiety-like behavior after stress. Using flow cytometry analysis, surface expression of several inflammatory markers was measured, including CD14, TLR4, CD86, and MHCII. Stress exposure increased the expression of CD14, TLR4, and CD86 on microglia, while only CD86 was increased on non-microglial macrophages. Pharmacological blockade of beta-adrenergic signaling during stress prevented the increased expression of CD14 on microglia. Propranolol administration also prevented increases in Iba1 staining in the medial amygdala, PFC, and hippocampus, but not PVN. These data suggest that chronic stress-mediated changes in microglial receptor expression and morphology are correlated with the emergence of anxiety-like behavior and corresponding neuronal activity in mice.

A study by Poggini et al. investigated the effects of minocycline, a tetracycline antibiotic that has inhibitory effects on microglia, on the behavioral, electrophysiological, and histological effects of chronic mild stress exposure in male mice [95]. Minocycline administration immediately following stress augmented hippocampal CA1 long-term potentiation (LTP) compared to vehicle-injected animals. Furthermore, when assessed three weeks following the stress period, mice that received minocycline showed no differences in microglia cell number or morphology in prefrontal cortical and hippocampal regions. Together, these data demonstrate that minocycline treatment improves hippocampal function following chronic stress. However, the role of microglia in this protection remains unclear. This study did not include experimental groups that did not receive stress, which prohibited comparison between stressed and non-stressed groups. Of note, cells selected for analysis were both Iba1+ and Tmem119+. This approach allowed for a more precise selection of microglia relative to the use of an Iba1+ stain alone, which might also identify non-microglial brain macrophages. There is evidence that peripheral macrophages, which also express Iba1, may be recruited into the brain parenchyma following stress [58]. Trafficked monocytes exhibit spatial selectivity, arriving in stress-activated brain structures, including the hippocampus [59]. Differences in stress paradigm and variation in immunofluorescence-targeting approach may explain differential results regarding microglial morphology changes in the hippocampus following chronic stress.

A recent study by Yuan et al. examined the effect of corticosterone application directly onto the central nucleus of the amygdala (CeA) [74]. Specifically, these studies aimed to understand the impact of chronic glucocorticoid signaling on amygdala regulation of visceral hypersensitivity, a hallmark of stress-related irritable bowel syndrome. Seven days of corticosterone exposure via an intracranial steroid pellet resulted in upregulation of microglial C1q and complement receptor 3 (C3R) expression in the CeA. Further studies reported an increase in microglial synapse engulfment when utilizing combined RNAscope, qPCR, and immunofluorescence approaches. Critically, the authors demonstrated that microglia-mediated effects of corticosterone exposure could be mitigated through treatment with either minocycline or neutrophil inhibitory factor (NIF). In total, these experiments provide evidence of a direct link between elevated glucocorticoid signaling and complement system regulation of microglia in promoting stress-related remodeling. 

The basolateral amygdala (BLA) is a key mediator of stress-related behavioral effects, particularly the emergence of anxiety-like symptoms. Interestingly, chronic stress promotes dendritic spine growth in BLA principal neurons [57]. Work by Bollinger et al. describes the sex- and stress modality-dependent activation of immune factors and microglial morphology in mesolimbic nodes, including BLA [70]. The authors demonstrated that exposure to chronic stress has differential effects on BLA gene expression in male and female mice. Chronic stress led to selective upregulation of inducible nitric oxide synthase (iNOS) in females, selective decreases in CD40 and Arg1 expression in females, and an increase in CD200R in males only. Microglia cell number did not differ across sex and stress comparisons. These results demonstrate that chronic stress perturbs microglial signaling in a sex-specific manner in BLA.

## 5. Microglia Respond to Psychostimulant Drug Exposure 

**Figure 2:**
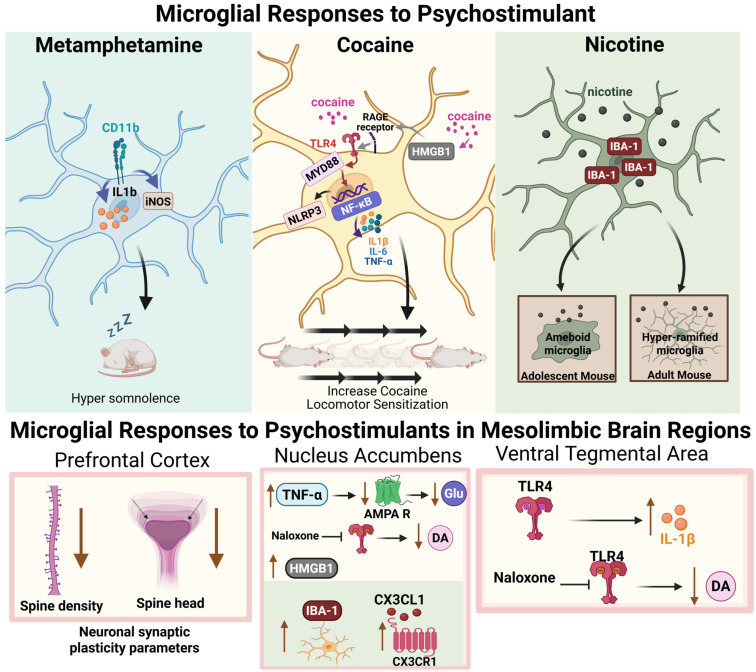
**Overview of psychostimulant effects on microglia across brain regions.** This figure illustrates the key mechanisms discussed in Section 5 of the review, summarizing the effects of methamphetamine, cocaine, and nicotine on microglia (upper panel) and mesolimbic brain regions (lower panel). Panels are color-coded to match each drug with its associated effects: methamphetamine (blue), cocaine (yellow), and nicotine (green). **Upper panel—microglial effects. Left:** Methamphetamine increases CD11b expression in microglia, leading to elevated IL-1β levels and prolonged inducible nitric oxide synthase (iNOS) activity within CD11b^+^ cells [100]. Additionally, methamphetamine-induced hypersomnolence has been associated with CD11b^+^ microglial activation, as indicated by the blue arrows [101]. **Middle:** Cocaine activates microglia through multiple inflammatory pathways. Direct binding to Toll-like receptor 4 (TLR4) initiates MYD88 and NF-κB signaling (red arrow), resulting in increased IL-6, IL-1β, and TNF-α expression (purple arrow). NF-κB activation can also occur independently of TLR4. Cocaine also increases release of high-mobility group box 1 (HMGB1), which signals via the receptor for advanced glycation end products (RAGE), a receptor interacting with several TLRs [102]. Cocaine also engages in other inflammatory cascades, such as the NLRP3 inflammasome by the activation of nuclear receptors. Microglial activation has been shown to be essential for cocaine-induced behavioral sensitization [103]. **Right:** Nicotine increases IBA-1 expression in microglia. It has a developmental stage-dependent effect on microglial morphology: in adolescent animals, nicotine promotes an amoeboid morphology, while in adults, it induces a more ramified phenotype [104]. **Lower panel—brain region effects. Right (PFC):** Cocaine (yellow) reduces dendritic spine density and spine head diameter in the prefrontal cortex (PFC) [105]. **Middle (NAc):** Cocaine-induced TNF-α elevation in the nucleus accumbens (NAc) promotes AMPA receptor reinsertion, reducing glutamatergic signaling [106,107]. These effects are TLR4-dependent and are blocked by (+/−)-naloxone, which also reduces dopaminergic signaling. Cocaine also increases HMGB1, specifically in the NAc [102]. Nicotine (green) within the NAC induces an increase in IBA-1^+^ microglia in the NAc and upregulates CX3CL1–CX3CR1 signaling, which is essential for nicotine’s effects during adolescence [104,108]. The CX3CL1-CX3CR1 signaling was shown to be essential for the effects of nicotine in adolescence. **Left (VTA):** In the ventral tegmental area (VTA), cocaine effects are also mediated through TLR4, with (+/−)-naloxone again blocking TLR4 and dampening dopaminergic activity. Cocaine increases TLR4 expression in VTA microglia, elevating IL-1β and contributing to an inflammatory state [104]. Overall, the figure summarizes the main points of the review, showing how microglia is involved in different psychostimulants’ activity. Figure made using BioRender.com.

Recent studies demonstrated that microglia are responsive to psychostimulant drugs and may contribute to brain alterations following prolonged drug use (for review see: [96,97,98,99]. Corticolimbic remodeling following psychostimulant exposure has been extensively described, yet the specific role of microglia in these processes has not been elucidated and thus can only be proposed as a future direction of study. The next section will provide an overview of literature describing how microglia react to drug exposure, with particular attention paid to in vivo studies. The first section will describe what is known about neuronal remodeling in the absence of microglial investigation, while subsequent sections will detail how microglia respond to psychostimulants and may influence drug-related behaviors (See Figure 2 for overview of psychostimulant drug effects on microglia).

***Evidence for psychostimulant-induced mesocorticolimbic remodeling:*** Changes in neuronal structure and function following prolonged psychostimulant use have been well documented in preclinical and human studies. Neuroimaging data gathered from people who use cocaine show significant alterations in mesocorticolimbic functional connectivity, which may underlie alterations in motivated behavior at various stages of the addiction cycle [109,110]. Basic neuroscience sought to explain the molecular underpinnings of psychostimulant effects on neuronal structure within reward circuitry. Physical changes in dendritic spines, which represent both transient and long-lived features of the intercellular synaptic interface, emerged as observable markers of structural plasticity following chronic psychostimulant exposure.

Interestingly, multiple preclinical studies prior to the mid-2010s reported an increase in dendritic spine density after either self-administration or non-contingent psychostimulant experience in rodent PFC [111,112]. Work by Radley et al. eloquently distinguished cortical spine dynamics among multiple modalities of cocaine exposure, while employing a 3D morphometric approach to study spine morphology [113]. Previous studies relied largely on the Golgi–Cox staining method, which has several limitations, including the under-sampling of thin-type spines. Immature spines may be a particularly sensitive barometer of drug- or stress-related plasticity. The authors demonstrated that response-contingent (i.e., self-administered), but not yoked or experimenter-administered, cocaine exposure results in diminished apical spine density and increased spine head diameter measured in prefrontal cortical neurons after 2 weeks of abstinence [113]. Importantly, these observations suggest that these effects of cocaine in the rodent PFC are dependent on volitional drug use. Continued technological improvements allowed for greater appreciation of altered spine morphology following drug exposure; however, species-specific and temporal considerations may nonetheless complicate our interpretation of these findings [111,114]. 

The timepoint at which neuron structure is assessed following drug administration, as well as the modality of drug exposure, led to differential reports of PFC spine density. Work by Siemsen et al. describes the “biphasic” effect of cocaine self-administration and subsequent abstinence on the spine dynamics of pyramidal neurons in PFC that project to NAc [105]. This glutamatergic projection has been well characterized as an important mediator of drug-seeking behavior. The authors find that immediately after a 2-week period of cocaine self-administration, prelimbic PFC spines undergo spine head shrinkage and reductions in measures of cellular activity, while the opposite is observed after one week of drug-free abstinence.

The NAc core is an important hub containing medium spiny neurons that are receptive not only to glutamate, but also to the modulatory efforts of dopamine, which coalesce to regulate motivated behavior. Psychostimulants rapidly evoke plasticity in NAc medium spiny neurons, resulting in sustained increases in spine density [115]. Although the duration and relevance of drug-mediated neurostructural changes have been debated, an increase in spine density in the NAc core may indicate increased synaptic transmissions from the medial PFC, VTA, CeA, or BLA that are important for sustained drug-related behaviors.

The role of microglia in mediating changes in spine dynamics after psychostimulant exposure is largely unknown. However, as mentioned previously, studies of chronic stress-related dendritic spine alterations correlated with microglial activity such as complement-mediated engulfment. Future studies are needed to elucidate the role of microglia in effecting changes in neuronal morphology after psychostimulant exposure.

***Microglia are sensitive to psychostimulant exposure:*** Microglia are innate immune cells that constantly survey the brain parenchyma. Commonly misused psychostimulants, including cocaine and methamphetamine, are sensed by microglia as xenobiotics (i.e., foreign materials) that trigger inflammatory processes through activation of the Toll-like receptor 4 (TLR4). TLR4 is a member of a family of pattern recognition receptors (PRRs) that evolved to respond to molecular cues associated with cellular damage. When the canonical endotoxin, lipopolysaccharide (LPS), is introduced to microglia, activation of TLR4 leads to pro-inflammatory signaling through MyD88-mediated regulation of NFκβ expression, including the production of cytokines such as TNF-α, interleuken-1β (IL-1β), and interleuken-6 (IL-6).

Through a series of experiments exploring the effect of naloxone and naltrexone enantiomers (μ-opioid receptor antagonists) on glial (presumably microglial) activation in the VTA in response to morphine, it was discovered that cells are sensitive to the effects of naloxone in a non-stereo-specific manner [116]. Both the opioid-sensitive enantiomer, (−)-naloxone, and the opioid-insensitive (+)-naloxone inhibit glial activation by morphine, suggesting that morphine may produce opioid receptor-independent activation of microglia. Further work linked opioid action on glial cells to activation of TLR4 and its accompanying MD2 constituent [117,118]. Importantly, these findings have been extended to other commonly misused drugs, including alcohol and cocaine. Activation of TLR4 by cocaine may also contribute to the reinforcing effects of the drug, as well as the potential for relapse-like behavior after cessation of drug use [119,120].

In addition to its putative TLR4 activation, cocaine exposure has been shown to elicit neuroimmune signaling between neurons and microglia in limbic regions. High-mobility group box 1 (HMGB1) is a nuclear protein that can be released from cells and recognized as a damage-associated molecular pattern (DAMP). Microglia express receptors capable of binding HMGB1, including the receptor for advanced glycation end products (RAGE), which has a functional interaction with several TLRs. Recent studies demonstrated that HMGB1 signaling is important for the consolidation of cocaine-associated memories in rats. Ye and colleagues demonstrated that non-contingent cocaine exposure in rats leads to increased HMGB1 secretion by neurons in the NAc core [102]. Furthermore, the blockade of HMGB1 release using carbenoxolone (i.p.), a pannexin-1 channel blocker, prevents cocaine-induced conditioned place preference. The use of glycyrrhizin (i.p), which binds to HMGB1 protein to inhibit its activity, also attenuated place preference. Moreover, the authors demonstrated that minocycline, a putative microglial inhibitor, also blocked cocaine-conditioned place preference. Interestingly, treatment of rats with minocycline did not attenuate the cocaine-induced increase in HMGB1 levels.

Another inflammatory cascade recruited by cocaine exposure is the NLRP3 inflammasome. In a series of experiments, Chivero et al. describe NLRP3 induction in response to cocaine in both a microglia cell line and primary microglia culture as well as in mice receiving non-contingent cocaine injections [121]. In vitro studies revealed a dose-dependent increase in NRLP3 protein in response to cocaine application, while an increase in mature/active IL-1β was also reported for up to 12 h, which may be mediated by the microglial sigma receptor (sigma-1R). Mouse studies corroborated the effects shown in culture such that cocaine injections augmented the expression of NLRP3, Cd11b, ASC (apoptosis-associated speck-like protein containing a CARD; a NLRP3 mediator), and IL-1β, among other mediators, in striatal tissue. Finally, the authors showed that humans who experienced chronic cocaine dependence display similar post-mortem alterations, including increased IL-1β, ASC, Cd11b, and Iba1, albeit in cortical tissue. These studies point towards a mechanistic interaction between cocaine and microglia, via the sigma-1 receptor, to mediate NLRP3-dependent inflammatory signaling.

Another avenue by which microglia may impact brain dynamics is through the regulation of neurotropic factors, including brain-derived neurotrophic factors (BDNFs). A study by Cotto and colleagues suggests that microglial methyl CpG-binding protein 2 (MeCP2) may be important for BDNF regulation in the context of cocaine self-administration in rats [122]. The authors reported that 6 h of daily cocaine self-administration, but not 2 h of self-administration or saline control conditions, increased microglia (Iba1+ cells) in the frontal cortex and hippocampus. Additionally, they showed an increase in microglia cell body size in the frontal cortex, hippocampus, and NAc, suggesting a more ameboid orientation. The investigators also present evidence of increased MeCP2 and BDNF protein levels after 6 h daily cocaine access. Further studies demonstrated that MeCP2 expression and phosphorylation increase after cocaine exposure, resulting in its translocation from the nucleus in microglia, but not neurons, leading to decreased negative transcriptional regulation of BDNF by MeCP2 in microglia after cocaine. These data provide mechanistic insight into how microglia may respond to volitional cocaine administration to augmented BDNF levels associated with drug-induced plasticity.

Cocaine self-administration effects on microglia morphology in mesocorticolimbic regions have been further examined by Burkovetskaya and colleagues, who demonstrated that cocaine self-administration leads to an increase in Iba1 intensity and pro-inflammatory gene expression in the striatum, but not the PFC of cocaine self-administering mice [123]. Furthermore, the authors demonstrated that microglial morphology in the NAc was altered following cocaine self-administration, with notable reductions in branch length and complexity. These measurements were performed one day after the final cocaine self-administration session. Alterations in PFC spine morphology have been described after a period of abstinence [113], and accompanying changes in microglia might be expected. Further studies evaluating microglial signaling following a period of abstinence are needed to elucidate the role of cortical microglia for drug-related plasticity.

Methamphetamine self-administration in rats has been shown to alter cortical and striatal microglia populations both acutely and chronically [100]. Kays et al. examined the transcriptional signature of microglia following a methamphetamine “binge” in rats. They found that microglia shifted towards a reactive state two hours following a methamphetamine binge, characterized by increases in *Il1b*, but not other canonical markers of inflammation such as (*Il6*, *Tnf*, *Ccl2*, *iNos*, and *Nox*) [100]. Cortical changes (methamphetamine vs. saline) were completely abolished 3 days following drug exposure, while striatal changes were persistent but greatly reduced. These data extend finding involving cocaine to demonstrate that methamphetamine self-administration can also influence microglia activity in a region-specific manner in the mesocorticolimbic system.

***Microglia impact behavior following psychostimulant exposure:*** Interest in the idea that microglia may contribute to drug-related behaviors increased in recent years. However, most studies remain correlative, and the exact mechanisms through which microglia might mediate the effects of psychostimulants on the brain remain elusive.

Although its translational value is limited, testing for behavioral sensitization to the locomotor effects of cocaine and other psychostimulants is often a “first-stop” approach in many lines of investigation attempting to understand how commonly misused drugs impact reward circuitry. In rodents, repeated cocaine or amphetamine administration results in a progressive increase in locomotor responding with each subsequent experience. While its mechanism of action is unclear, the microglial inhibitor, minocycline, is often used to interrogate the in vivo effects of microglia. Chen et al. reported the effects of minocycline administration on cocaine-induced locomotor sensitization in male mice [103]. The authors demonstrate that while minocycline has no effect on acute cocaine-induced locomotor activity, it prevents locomotor sensitization to repeated cocaine injections. Although inhibition of microglial activity was assessed, the data suggest that microglia may play a role in addiction-related psychostimulant-induced neuroadaptations.

Lewitus and colleagues examined the effects of cocaine on microglia in the NAc and how microglial cytokine release impacts behavioral sensitization to cocaine [106]. They demonstrated that repeated cocaine delivery, but not a single injection, leads to increases in TNF-α mRNA and protein when measured 24 h following the terminal injection. Using a TNF-α knock-out mouse model, they found that disrupted TNF-α signaling potentiated locomotor activity after repeated cocaine injections and after a cocaine challenge following a period of abstinence compared to wild-type mice. Additionally, the authors showed that interruption of TNF-α signaling, specifically during the sensitization phase (i.e., period when animals were receiving daily cocaine injections) increased the locomotor response to cocaine. These data suggest that intact TNF-α signaling during cocaine exposure may buffer cocaine’s effects. The role of microglia in this process is supported by an experiment showing that deletion of TNF-α from CX3CR1-positive cells produced a similar potentiation of cocaine-induced locomotor activity [106]. In a previous study, Lewitus and colleagues showed that TNF-α attenuates glutamatergic synaptic strength in the NAc through removal of calcium-permeable AMPA receptors [107]. Together, these data suggest that TNF-α acts to reduce glutamatergic signaling that would otherwise drive the heightened NAc signaling that is necessary for sensitization. 

Other studies found that microglia can regulate cocaine self-administration. Linker et al. demonstrated microglial involvement in the effects of nicotine on cocaine self-administration [108]. The authors investigated the consequences of a history of nicotine exposure on cocaine self-administration behavior in adolescent and adult mice. They reported that adult mice that had a 4-day history of nicotine exposure (non-contingent, intravenous) showed no differences in cocaine self-administration (single-session) compared to animals pre-treated with saline. However, adolescent mice (age = P32) that were exposed to nicotine displayed enhanced cocaine-self administration. The adolescent mice pre-treated with nicotine also showed increased numbers of microglia (IBA1+ cells) in the NAc and BLA compared to adolescents pre-treated with saline. No differences in adult microglia number were reported between the two conditions. Analysis of IBA1 expression (fluorescence intensity) showed a decrease in NAc IBA1 levels in adult mice that received nicotine, but augmented levels in adolescent mice that received nicotine. There was significantly more IBA1 signal observed in the BLA of adolescent mice that received nicotine versus saline controls, yet no differences in BLA IBA1 expression were found in adult mice. Morphological analyses revealed a dichotomous effect of age in microglial phenotypes. For example, nicotine exposure in adolescent mice skewed microglia towards a more ameboid orientation (decreased process length, decreased process branching, and increased process diameter), while nicotine exposure in adult mice promoted an opposite phenotype (more ramified). To show the necessity of microglia for the effects of nicotine on increased adolescent cocaine-taking behavior, the investigators employed a pharmacological approach, using minocycline or PLX3397 throughout the experimental period to block microglial function. Treatment with minocycline abolished the increases in cocaine taking previously observed with nicotine exposure, including a significant reduction in NAc IBA1+ cells and attenuation of increased IBA+ cells in the BLA. Similar behavioral changes were reported in response to PLX treatment, although treatment with PLX resulted in microglial clearance from both NAc and BLA. Transcriptional analyses indicated that nicotine exposure increased the levels of both CX3CR1 and CX3CL1 in the adolescent NAc. Use of an RNAi tool to knock down CX3CL1 expression in the NAc blocked nicotine effects on cocaine self-administration in adolescent mice. These experiments demonstrate that microglia, through CX3CL1-CX3CR1 interactions, mediate the effects of nicotine exposure on cocaine-taking behavior. Evidence suggests that adolescent nicotine exposure may also impact motivation for cocaine in adulthood [104], but the role of microglia in this mechanism remains unclear.

As mentioned previously, microglia detect cocaine and other commonly misused drugs as xenobiotics, which can recruit TLR4-dependent signaling. In a study by Northcutt et al., the relationship between canonical immune signaling through TLR4 and the rewarding properties of cocaine was examined [104]. The authors demonstrated that isolated neonatal rat microglia displayed upregulated *Il1b* expression in response to cocaine application. This effect could be blocked by application of (+)-naloxone, a TLR4 antagonist. In vivo experiments revealed that *Il1b* expression was significantly increased in the VTA, but not NAc or PFC, when measured 30 min or 2 h after the cocaine injection. Again, the authors demonstrated that this effect was attenuated by administration of (+)-naloxone. Subsequent experiments showed that (+)-naloxone treatment blunted NAc dopamine levels following an i.p. injection of cocaine, an effect that was mimicked by administration of intra-VTA LPS-RA, a traditional TLR4 antagonist, or IL-1ra, an IL-1 receptor antagonist. These data indicate that TLR4 signaling following a cocaine injection plays a role in regulating dopamine dynamics in the VTA. The role of TLR4 was explored further in cocaine-conditioned place preference and self-administration studies. The authors report that (+)-naloxone or minocycline treatment prevent the development of cocaine-conditioned place preference in rats. In accordance with their findings using the conditioned place preference task, the authors reported that rats trained to self-administer cocaine consumed less drug when pretreated with (+)-naloxone. Additionally, studies using mice expressing an inactive mutant TLR4 showed that functional TLR4 is necessary for the maintenance of cocaine self-administration. Taken together, these data suggest that TLR4, localized on microglia, is a critical mediator of the rewarding and motivational properties of cocaine.

A related study by Brown and colleagues suggests that TLR4 signaling in VTA may also be important for the expression of cocaine-primed reinstatement behavior [119]. The authors observed that, after a period of cocaine self-administration and extinction, the TLR4 antagonist, LPS-RS, micro-infused into the VTA, attenuated cocaine-primed reinstatement of drug seeking in male rats. Importantly, this intervention had no effect on the motivation to consume sucrose after a period of self-administration/extinction. Additionally, the authors demonstrated that antagonism of IL-1 receptors in the VTA also attenuated cocaine-primed reinstatement. These findings demonstrate that TLR4 activity and IL-1 signaling in the VTA can selectively modulate drug seeking without affecting the seeking of a non-drug reinforcer.

A subsequent publication by Brown et al. explored the effects of continuous administration of (+)-naltrexone, a TLR4 antagonist, via osmotic minipump on the acquisition and maintenance of cocaine self-administration and the extinction and reinstatement of cocaine-seeking behavior [124]. The authors reported that (+)-naltrexone delivery during the self-administration period had no effect on drug taking or subsequent extinction or cue- or drug-primed reinstatement of cocaine seeking. Similarly, acute pretreatment with (+)-naltrexone failed to alter cocaine self-administration under a progressive ratio schedule. Next the authors examined the effects of TLR4 antagonism during a period of abstinence following chronic cocaine self-administration. There were no effects of TLR4 antagonism on cocaine cue seeking after 14 days of abstinence. However, acute pre-treatment with i.p. (+)-naltrexone attenuated cocaine-primed reinstatement. Site-specific testing revealed that the TLR4 antagonist LPS-Rs micro-infused into the NAc shell, but not the NAc core, attenuated cocaine-primed drug seeking. These data suggest that TLR4 signaling may have site-specific actions that influence cocaine seeking upon cocaine re-exposure following a period of drug abstinence.

Methamphetamine is a powerful psychostimulant with a well-established ability to alter circadian behaviors such as sleep/wake cycles. Cessation of methamphetamine use often results in a withdrawal period marked by hypersomnolence. The ability of microglia to mediate arousal states resulting from psychostimulant exposure was explored in a paper by Wisor and colleagues [101]. Using a ganciclovir-inducible transgene strategy in mice, the authors ablated CD11b+ cells in mice and tested for the effects of methamphetamine on sleep/wake behavior. Mice that were treated with ganciclovir (CD11b cell ablated) had a shorter latency to consolidated sleep compared to mice with intact CD11b+ cell function when given a relatively high dose of methamphetamine (2 mg/kg). Further molecular characterization revealed that methamphetamine promoted nitric oxide synthase (NOS) activity in both CD11b+ and CD11b− cells; however increased NOS activity was prolonged CD11b+ cells (2 h vs. 1 h). Cytokine expression was altered after methamphetamine exposure in CD11b+ cells only, which showed an increase in *Il1b*, but a decrease in *Tnf* transcripts. Notably, cells in these studies were isolated using CD11b+ microbeads and a magnetic separation protocol from whole brain isolates. Psychostimulants are known to produce region-specific alterations in cytokine expression that may be important for interpreting effects on neuronal function. These data provide evidence that microglia may be important mediators of the psychostimulant effects on arousal state.

## 6. Chronic Stress Effects on Drug Seeking: Potential Microglia Involvement

Although stress has been implicated in SUDs, the exact mechanisms that drive this relationship in humans remains elusive [2,125]. Preclinical studies investigated the effects of chronic stress on drug intake and drug seeking by utilizing rodent models and various stress protocols. Multiple mechanisms through which stress alters drug-taking/seeking behaviors have been proposed and will be discussed below. We will address these studies according to the temporal nature of stress exposure (i.e., stress exposure before/during or after drug experience). This is relevant because distinct mechanisms have been implicated in either stress promoting drug acquisition and intake versus stress that might influence relapse-like behavior. Finally, we will continue to explore the idea that microglia may play a role in chronic stress-mediated alterations in drug-related behavior in light of recent publications.

***Chronic stress promotes vulnerability to psychostimulant self-administration:*** In the early 1980s, Antelman et al. first described behavioral “cross-sensitization” between stressors and amphetamine [126]. They found that male rats that underwent repeated tail pressure stress exhibited increased sniffing behavior akin to rats that had undergone sensitization as a result of repeated amphetamine injections when given a subsequent amphetamine challenge. Interestingly, the group also demonstrated that a prior injection of amphetamine increased behavioral responses to tail pressure stressors, demonstrating that stress or drug experience can increase behavioral reactivity to each other (i.e., cross-sensitize). Studies by Piazza et al. [127,128,129], took this concept one step further by demonstrating that the acquisition of amphetamine self-administration is dependent upon both (1) reactivity to a stressor (i.e., novelty induced activity) and (2) prior drug experience. For example, the authors reported that rats who displayed higher levels of locomotion when exposed to a novel context were also more likely to self-administer amphetamine compared to those who display lower levels of locomotion in a novel context [127]. Yet, when rats exhibiting low levels of novelty-induced behavior receive a repeated amphetamine injection regimen prior to self-administration, the difference in amphetamine self-administration between the high- and low-novelty responding groups is eliminated, suggesting that drug experience can act similarly to “trait-like” predisposition. Taken together, these two papers suggest that repeated stress and drug exposure can sensitize a rodent to the behavioral effects of psychostimulants, while also raising the probability that they will self-administer the drug. Moreover, these reports demonstrate that pre-existing traits, such as a behavioral response to novelty, may interact with drug exposure to influence drug-taking behaviors.

Relatedly, Mantsch et al. described in further detail the phenomenon of high- and low-responders to novelty in their susceptibility to cocaine self-administration [130]. Utilizing a long-access (6 h) model of daily cocaine access, the authors show that the difference in cocaine self-administration acquisition between the two groups based on novelty response only exists when the dose of cocaine offered is relatively low (0.25 mg/kg/inf). Interestingly, if rats are offered higher doses of cocaine, the difference between high- and low-responders is eliminated.

Work describing the effects of repeated stress exposure on subsequent vulnerability to drug self-administration continued through the last 40 years. The action of corticosterone, the major circulating stress hormone in rodents, has often been the focus of investigation. An understanding of the effector and regulatory properties of corticosterone in the stress response is helpful when considering the impact of chronic stress effects on the brain.

The hypothalamic–pituitary–adrenal (HPA) axis is activated following stressor onset, but also according to circadian and ultradian rhythms, and culminates in the secretion of corticosterone from the inner layers of the adrenal cortex. Corticosterone regulates the activity of the HPA axis via a negative feedback loop, whereby elevated corticosterone inhibits the secretion of corticotropin releasing factor (CRF) and regulates gene transcription, leading to cessation of HPA activity. Exposure to repeated stressors or exogenous corticosterone supplementation leads to dysregulation of HPA axis balance due to the decreased sensitivity to CORT that is needed to terminate HPA axis activity. Endogenous corticosterone release is pulsatile, and chronically elevated levels impair nuclear translocation of the glucocorticoid receptor (GR). The inability of corticosterone to effectively exert negative feedback through GR may contribute to cellular and behavioral consequences of chronic stress exposure.

Piazza et al. conducted a series of experiments showing that the duration of corticosterone secretion following a stressor is positively correlated with the likelihood of later amphetamine self-administration [129]. Later, Deroche and colleagues showed that corticosterone secretion following repeated stressors is necessary for the subsequent facilitation of amphetamine self-administration, such that animals that underwent adrenalectomy and corticosterone replacement failed to show stress-induced facilitation of drug self-administration [131]. To determine sufficiency in addition to the necessity of corticosterone signaling, Deroche et al. later demonstrated that corticosterone supplementation is sufficient to produce behavioral sensitization to amphetamine [132]. 

These observations were extended to cocaine self-administration in rats through the work of Goeders and colleagues, who demonstrated that non-contingent electric foot shock (EFS) exposure promoted the acquisition cocaine self-administration when compared to unstressed animals [133]. Levels of circulating corticosterone were associated with drug-taking behavior in this study, suggesting that stress-induced corticosterone signaling plays a permissive role in psychostimulant self-administration. The ability of stress to promote the acquisition of cocaine self-administration has also been demonstrated using a variety of other stressors, including repeated restraint, food deprivation, social isolation, witnessing a conspecific receiving shock, and social defeat [134,135,136,137,138]. Moreover, it was demonstrated that administration of corticosterone, at a dose that produces blood levels comparable to those observed with EFS, also promoted the acquisition of self-administration assessed using a similar protocol [139].

Social stress is a complex challenge to rodent homeostasis and can invoke robust activation of the HPA axis. Covington et al. were among the first to demonstrate that social defeat stress leads to behavioral sensitization to both cocaine and amphetamine [140]. Additionally, animals that were sensitized to stress or cocaine achieved greater drug intake in a 24-h “binge-like” model of cocaine self-administration. These data suggest that social stress exposure is sufficient to produce relevant alterations in drug-taking behavior such as that of repeated psychostimulant exposure (for review see [141]).

In mice, the relationship between chronic stress exposure and augmentation of cocaine self-administration appears to be nuanced. For example, Engeln et al. investigated the effect of housing conditions and chronic social defeat stress on cocaine intake [142]. Interestingly, the authors demonstrated that susceptibility to stress, defined based on social avoidance behavior, had a bidirectional effect on cocaine intake that was dependent on housing conditions. Pair-housed animals that exhibited increased social avoidance after stress exhibited reduced cocaine intake, while single-housed animals that displayed increased social avoidance after stress showed an increase in cocaine intake. These results bolster the argument that the social environment is consequential when considering the interactions of stress and drug use.

Stress related to the drug taking environment has also been investigated as a mediator of increased psychostimulant intake in preclinical models. Since features including escalation of drug intake or resistance to punishment in animal models may highlight relevant behavioral phenotypes observed in human populations, it is important to understand how stress and CORT signaling may influence these behaviors. Mantsch et al. describe an intra-session electric foot shock (EFS) paradigm designed to potentiate CORT signaling in a short access (<2 h) cocaine self-administration session that would otherwise produce stable drug taking patterns [143]. Interestingly, the authors demonstrate that non-contingent exposure to a mild stressor, EFS, is sufficient to potentiate cocaine-taking behavior, resulting in potentiation of inter-session drug intake akin to that observed in extended access models [144,145,146].

***Chronic stress promotes drug-seeking behavior:*** In the above section, we discussed the roles of chronic stress and glucocorticoid signaling in the acquisition and maintenance of psychostimulant self-administration. The next section will review the effects of chronic stress on drug-seeking behavior during or after a period of abstinence. It should be noted, however, that what is often referred to as stress-induced drug seeking (i.e., relapse-like behavior or stress-induced reinstatement) typically refers to drug seeking that is precipitated by a bout of acute stress. In this section, we will focus on studies examining the impact of chronic, rather than acute, stressors on drug-seeking behaviors. 

A study by Yu et al. demonstrated that rats with a previous history of nicotine self-administration show increased nicotine consumption when they resume self-administration following a period of abstinence during which they received repeated restraint [147]. Interestingly, the increase in nicotine intake resulting from stress exposure was only evident in rats that received at least four sessions of restraint stress. 

Drug-seeking behavior has been shown to increase in a time-dependent manner during abstinence from psychostimulants, a phenomenon that has been termed “incubation of craving”. Chronic stress exposure during abstinence has been shown to increase the rate of incubation, especially during early phases. Glynn et al. utilized an extended access model of daily cocaine self-administration followed by forced abstinence to investigate the role of stress during early withdrawal on later cue-induced drug seeking [148]. They found that rats in their study increased cocaine seeking in a time-dependent manner (i.e., after a two-week abstinence period); however, rats that were subjected to repeated restraint stress exhibited heightened drug-seeking behavior. The mechanism through which chronic stress exposure during early abstinence influences drug-seeking behavior may involve altered neuronal activity in the basolateral amygdala as measured using in vivo extracellular single-unit recording in anesthetized rats [149].

Although microglia actions have not been specifically linked to the changes in neuronal function following stress during abstinence from psychostimulants, they have been shown to play a role in the neuroadaptations driving increased drug seeking during the abstinence period. Reverte et al. showed that microglia play a vital role in neuronal alterations in the NAc shell following cocaine exposure in mice [150]. In this study, mice were trained in a conditioned place preference paradigm using non-contingent injections of cocaine. During an abstinence period of 20 days, mice were fed either a standard diet or a diet supplemented with PLX5622 (PLX), a CSF1R inhibitor. Both mice that received standard or PLX-containing chow exhibited an increased preference for the cocaine-paired chamber when compared to mice who received saline in both chambers, meaning that PLX supplementation did not disrupt the conditioned rewarding effects of cocaine. However, when the experimenters quantified the locomotor activity during preference testing, cocaine-treated mice that received standard chow showed augmented locomotor motor activity, while PLX appears to block this effect of cocaine exposure. The authors also describe cellular changes following repeated cocaine exposure, including increased dendritic spine density in the NAc shell. Following 21 days of abstinence, microglia in the cocaine-exposed mice displayed a decrease in the number of branches, total branch length, arborization domain, and microglial domain, while showing an increase in morphological index (ratio of soma volume and arborization domain). Additionally, the authors reported an increase in NAc shell neuronal spine density and spine head diameter, both of which are blocked by treatment with PLX during the abstinence period. Whole-cell electrophysiological recording demonstrated that cocaine exposure led to an increase in rectification index, which is also blocked in mice treated with PLX, supporting the hypothesis that microglia contribute to the structural and functional changes associated with abstinence from cocaine.

***Microglia may facilitate the effects of chronic stress on motivation for psychostimulants:*** As described above, the effects of chronic stress on drug taking and seeking have been thoroughly investigated. However, the cellular and molecular bases for stress-related changes in motivation for psychostimulants remain incompletely understood and neuroimmune involvement is likely.

Recently, scientists have begun to probe the pathophysiology of stress-related disorders through the lens of neuroimmune interactions. The immune system functions in concert with neurons and other brain cells to maintain homeostasis and influence behavior. The actions of microglia have been implicated in mediating the effects of chronic stress on neuronal structure and the emergence of depression- or anxiety-like behaviors in rodents. Thus, microglia help create a “brain stress” state through which other factors, including psychostimulant use, might produce more severe changes in behavior.

Only a few studies to date directly investigated the role of microglia in mediating stress-related changes in response psychostimulant use or non-contingent exposure. Kelly et al. describe the effects of chronic corticosterone exposure in mice that would later receive a non-contingent injection of methamphetamine [151]. The rationale for pre-treating the mice with chronic corticosterone was to leverage the canonical anti-inflammatory properties of corticosterone to limit the levels of neuroinflammatory markers associated with methamphetamine-induced neurotoxicity. Interestingly, the authors found that chronic pre-treatment with corticosterone markedly increased levels of neuroinflammatory signals (IL-1β, LIF, and CCL-2) when combined with a methamphetamine injection. These unexpected data suggest that chronic corticosterone may sensitize rodents to the neuroinflammatory consequences of methamphetamine exposure.

Earlier, we discussed the relevance of early life stress in shaping drug-related motivated behaviors in adulthood. A study by Lo Iacono et al. describes the effects of early life stress (PND 14-22) on subsequent vulnerability to cocaine-seeking behavior in male and female mice [152]. Utilizing a social stress procedure, the authors demonstrated that social stress during early life led to microglial alterations in the VTA. Interestingly, the number of microglia cells in the VTA were unchanged after social stress alone, but when combined with non-contingent cocaine injections, microglia cell number was increased compared to non-stressed conditions. Mice that underwent social stress also showed increases in microglia soma size. Furthermore, the social stress/cocaine exposure condition showed the highest values in Scholl analyses considering intersections, length, and number of nodes. It is worth noting that not only did microglia in stress/cocaine mice exhibit increased soma size, which commonly infers a transition to an ameboid orientation, but they also showed greater branching according to Scholl analysis metrics, suggesting a hyper-ramified state. These morphological findings tend to conflict with canonical descriptions of microglial states (e.g., ramified vs. ameboid is to surveillant vs. activated), which underscores the importance of detailed morphological markers and non-binary classification schemes when defining microglial activity. Additionally, the authors report that cocaine-conditioned place preference expressed by mice that underwent social stress exposure in early life could be blocked by minocycline treatment, suggesting that microglia may facilitate changes in reward circuity that result in expression of cocaine preference in adulthood after early life stress.

Perhaps the most promising data supporting the hypothesis that microglia play a role in the cellular and behavioral adaptations following stress exposure and psychostimulant use come from a recent paper by Avalos et al. [82]. In this study, male rats underwent chronic restraint stress (CRS) or a sham treatment and then underwent cocaine self-administration. During the self-administration phase, half of the rats received daily minocycline, a putative microglial inhibitor. Results show that CRS prior to cocaine self-administration results in an escalation of cocaine intake over the course of ten days. Interestingly, treatment with minocycline during the self-administration period blocks these effects of CRS, limiting cocaine intake to the level of non-CRS animals. Importantly, the authors also demonstrate that CRS or minocycline treatments have no effect on sucrose intake in a separate cohort of animals, suggesting that the potentiating effects of CRS are specific to cocaine intake. Histological analyses revealed that NAc core microglia exhibit hyper-ramified morphological markers in the CRS/VEH animals only. Minocycline treatment blocked the effects of CRS on the altered morphology of NAc core microglia. Additionally, NAc core neuronal spine density was increased as a result of CRS exposure prior to cocaine self-administration, while this increase was blocked by treatment with minocycline. Molecular studies showed that the CRS animals exhibited an increase in NAc expression of *tnf-α* transcripts, an increase that was once again blocked by treatment with minocycline. These studies provide compelling evidence that microglia are critically involved in stress-related increases in cocaine intake, along with molecular and cellular adaptations resulting from dual exposure to psychostimulants and chronic stress.

## 7. Conclusions

Throughout this review, we discussed emerging data describing the neurobiological and behavioral impacts of microglia action in the context of stress and/or psychostimulant exposure. Readers who are inclined to better understand microglial physiology should consult other comprehensive reviews, including [153,154]. Comparing methods for analyzing microglial function highlights the importance of a multimodal approach when evaluating responses to pharmacological and environmental exposures. While transcriptomic and proteomic techniques offer valuable insight into gene expression and protein levels, they are often limited by technical constraints, however, morphological analysis, although indirect, remains a powerful and accessible tool for assessing dynamic, region-specific microglial changes. Ultimately, no single method can fully capture the complexity of microglial function.

Despite decades of research, new pharmacotherapeutic approaches for the management of SUDs are lacking. Progress awaits a better understanding of factors that differentiate patient subpopulations, thus enabling individualized treatment approaches. In many, stress is a key contributing factor to drug use, and the understanding that neuroimmune interactions mediated through microglia are determinants of the influence of stress in SUDs has great potential to guide promising new interventions. However, our understanding of how microglia regulate addiction-related neuronal function and neuroplasticity in the context of stress remains limited, and much research is needed to better understand this critical issue so that safe and effective strategies targeting microglia can be advanced.

## Data Availability

No new data were created or analyzed in this study. Data sharing is not applicable to this article.
